# Effects of Parsley (*Petroselinum crispum*) and its Flavonol Constituents, Kaempferol and Quercetin, on Serum Uric Acid Levels, Biomarkers of Oxidative Stress and Liver Xanthine Oxidoreductase Aactivity inOxonate-Induced Hyperuricemic Rats

**Published:** 2011

**Authors:** Fatemeh Haidari, Seid Ali Keshavarz, Majid Mohammad Shahi, Soltan-Ali Mahboob, Mohammad-Reza Rashidi

**Affiliations:** a *Nutrition Department of Para-Medical School, Ahvaz Jundishapur University of Medical Sciences, Iran.*; b *School of Health and Institute of Public Health Research, Tehran University of Medical Sciences, Iran.*; c *School of Health and Nutrition, Tabriz University of Medical Sciences, Iran.*; d *Biotechnology Research Center, Tabriz University of Medical Sciences, Iran.*

**Keywords:** Hyperuricemia, Oxidative stress, Quercetin; Kaempferol, Xanthine oxidoreductase

## Abstract

Increased serum uric acid is known to be a major risk related to the development of several oxidative stress diseases. The aim of this study was to investigate the effect of parsley, quercetin and kaempferol on serum uric acid levels, liver xanthine oxidoreductase activity and two non-invasive biomarkers of oxidative stress (total antioxidant capacity and malondialdehyde concentration) in normal and oxonate-induced hyperuricemic rats. A total of 60 male Wistar rats were randomly divided into ten equal groups; including 5 normal groups (vehicle, parsley, quercetin, kaempferol and allopurinol) and 5 hyperuricemic groups (vehicle, parsley, quercetin, kaempferol and allopurinol). Parsley (5 g/Kg), quercetin (5 mg/Kg), kaempferol (5 mg/Kg) and allopurinol (5 mg/Kg) were administrated to the corresponding groups by oral gavage once a day for 2 weeks. The results showed that parsley and its flavonol did not cause any significant reduction in the serum uric acid levels in normal rats, but significantly reduced the serum uric acid levels of hyperuricemic rats in a time-dependent manner. All treatments significantly inhibited liver xanthine oxidoreductase activity. Parsley, kaempferol and quercetin treatment led also to a significant increase in total antioxidant capacity and decrease in malondialdehyde concentration in hyperuricemic rats. Although the hypouricemic effect of allopurinol was much higher than that of parsley and its flavonol constituents, it could not significantly change oxidative stress biomarkers. These features of parsley and its flavonols make them as a possible alternative for allopurinol, or at least in combination therapy to minimize the side effects of allopurinol to treat hyperuricemia and oxidative stress diseases.

## Introduction

Hyperuricemia which is distinguished by abnormal high levels of uric acid is present in 5-30% of the general population and appears to be increasing worldwide (1). It has been considered as an important risk factor for gout and may be associated with development of several oxidative stress diseases such as cancer and cardiovascular diseases (2, 3). The control of uric acid production has been widely regarded as a key factor in the prevention and treatment of hyperuricemia (4). Liver xanthine oxidoreductase is the key enzyme in the catabolism of purines, and catalyses the oxidation of hypoxanthine to xanthine and xanthine to uric acid. This enzyme occurs in two distinct forms. Xanthine dehydrogenase (XDH) is the frequent active form under physiological conditions. Under pathological states, however, in parallel to the degradation of ATP into adenine and xanthine, an extensive conversion of XDH to xanthine oxidase (XO) takes place. The latter uses molecular oxygen as electron acceptor and leads to the formation of superoxide anion and hydrogen peroxide in parallel with uric acid production and acts as a source of reactive oxygen species (ROS) (5). Therefore, the inhibition of XO activity may decrease uric acid and ROS production, and results in anti-hyperuricemic and antioxidative effects.

Allopurinol is the only XO inhibitor under the clinical application and has served as a dominant uric acid-lowering agent in the past four decade (6). However, some severe adverse effects such as hepatitis, nephropathy and allergic reactions limit the clinical use of allopurinol (3). Several *in-vitro *observations confirmed the XO inhibitory activity of some phytochemicals such as flavonoids (7-10). Flavonoids are non-nutrient polyphenolic compounds that occur in plants and consist of six major classes based on specific structural differences: flavonols, flavones, flavanones, catechins, anthocyanidins, and isoflavones (11). Therefore, a promising approach for hyperuricemia and its complications might be a combination therapy utilizing dietary flavonoids and hypouricemic pharmaceuticals at a suboptimal dosage to minimize any potential adverse side effects.

Parsley (*Petroselinum crispum*) is a member of Apiaceous family that has been employed in the food, pharmaceutical, perfume, and cosmetic industries (12). Parsley (*Petroselinum crispum*) is widely distributed in Iran. In folk medicine, parsley (*Petroselinum crispum*) is used to treat a wide variety of conditions (13). As a traditional medicine for hyperuricemia and gout, parsley (*Petroselinum crispum*) has been used in Iran. Phytochemical screening of parsley (*Petroselinum crispum*) has revealed the presence of several classes of flavonoids (14). Flavonols (kaempferol and quercetin) and flavones (apigenin and luteolin), which occur as glycosidic form in nature, are major flavonoids found in parsley (*Petroselinum crispum*) and other apiaceous vegetables (15). Kaempferol and quercetin, which belong to flavonol group, possess a wide range of biochemical and pharmacological effects and have been recommended as chemopreventive agents or nutritional supplements (9, 11). The predominant mechanism of their biological actions is thought to result from antioxidant activity, enzyme inhibition, and the capacity to scavenge free radicals (7, 9). Therefore, it is speculated that the health promoting effect of parsley (*Petroselinum crispum*) may be due to its flavonol constituents and the content of flavonoid compounds in parsley is about 100 mg/100 g fresh weight (16).

The purpose of this study was to investigate the effects of oral administration of parsley (*Petroselinum crispum*) and its major flavonol constituents (kaempferol and quercetin) on serum uric acid levels, liver xanthine oxidase/dehydrogenase activity and two serum non-invasive biomarkers of oxidative stress (total antioxidant capacity and malondialdehyde concentration) in normal and oxonate-induced hyperuricemic rats.

## Experimental

*Reagent*

Quercetin, kaempferol, potassium oxonate, xanthine, nicotinamide adenine dinucleotide (NAD^+^), uric acid, allopurinol, tetraethoxypropane (TEP), trichloroacetic acid (TCA), 2-thiobarbituric acid (TBA) and bicinchoninic acid kit were purchased from Sigma-Aldrich Chemical Co. (Steinheim, Germany). All other reagents were purchased from Merck (Darmstadt, Germany). The reagents used were from of analytical grades. Parsley (*Petroselinum crispum*) leaves were collected from a same vegetable garden, Tabriz, Iran. The following study was conducted in the Department of Nutrition and Biochemistry, Tehran University of Medical Sciences, Iran, between May 2007 and October 2008. 

*Test compound preparation*

Parsley (*Petroselinum crispum*) leaves were carefully washed with water and left to dry at room temperature. Then they were weighted and completely blended in distilled water (1 : 1 w/v). Quercetin and kaempferol were dissolved in propylene glycol (17). Allopurinol used as a positive control, was prepared in 0.9% saline. All freshly prepared juicy samples were administrated to the corresponding groups by oral gavage once a day for 2 weeks.

*Animals*

A total of 60 male Wistar rats (body weights: 180-200 g) were obtained from the animal house of Tabriz University of Medical Sciences, Iran. They were fed with a commercial laboratory diet and allowed food and water ad libitum for an acclimatization period of 1 week prior to the experiment. All animals were maintained on a 12 h/12 h light/dark cycle and the temperature and humidity were kept at 18 ± 1°C and 50%, respectively. They were handled according to the recommendation of the local and national ethic committees.

*Animal model of hyperuricemia in rats*

Experimentally-induced hyperuricemia in rats (due to inhibition of uricase with potassium oxonate) was used to study antihyperuricemic and antioxidant effects of test compounds (16). Briefly, 250 mg/Kg, uricase inhibitor, potassium oxonate (PO), dissolved in 0.9% saline solution was administrated intraperitoneally to each animal 1 h before oral administration of test compounds.

*Exprimental design*

The animals were randomly divided into ten equal groups (n = 6). group 1: untreated, non- hyperuricemic animals; group 2: normal animals given 5 g/Kg parsley; group 3: normal animals given 5 mg/Kg kaempferol; group 4: normal animals given 5 mg/Kg quercetin; group 5: normal animals given 5 mg/Kg allopurinol; group 6: hyperuricemic animals; group 7: hyperuricemic animals given 5 g/Kg parsley; group 8: hyperuricemic animals given 5 mg/Kg kaempferol; group 9: hyperuricemic animals given 5 mg/Kg quercetin; group 10: hyperuricemic animals given 5 mg/Kg allopurinol.

*Sample preparation*

Blood sample was taken from each rat by cutting the tail tip 1 h after the test compound administration at first, 7^th ^and 14^th^ days of the study. Serum was obtained by centrifuging blood sample at 6000 rpm for 10 min. For HPLC analysis, the serum was filtered using SPARTAN 13/0.45 RC, Watman. The sera were stored at -20°C until use. At the end of the experiment, rats were anesthetized between 09.00 and 10.00 am. Their livers were removed, weighed and then rapidly washed in cold saline (0.9%) and placed in ice-cold isotonic potassium chloride solution (1.15% KCl w/v) containing 0.1 mM EDTA. The livers were then chopped into 4-5 volumes of 50 mM phosphate buffer (pH 7.4) and homogenized by a homogenizer fitted with a Teflon pestle. The homogenate was then centrifuged at 3000 g for 10 min, the lipid layer was carefully removed, and the resulting supernatant fraction was further centrifuged at 15,000 g for 60 min at 4°C. The supernatant was stored at -20°C until use. At the end of the experiment, rats were anesthetized between 09.00 and 10.00 am. Their livers were removed, weighed and then rapidly washed in cold saline (0.9%) and placed in ice-cold isotonic potassium chloride solution (1.15% KCl w/v) containing 0.1 mM EDTA. The livers were then chopped into 4-5 volumes of 50 mM phosphate buffer (pH 7.4) and homogenized by a homogenizer fitted with a Teflon pestle. The homogenate was then centrifuged at 3000 g for 10 min, the lipid layer was carefully removed, and the resulting supernatant fraction was further centrifuged at 15,000 g for 60 min at 4°C. The supernatant was stored at -80°C till the use time (18-20).

*Uric acid determination*

The serum uric acid levels were analyzed by the high performance liquid chromatography (HPLC) method using a system supplied by Waters Associates, Northwich, Cheshire which consisted of a Waters 515 pump, Waters 717 plus Autosampler, Waters 2487, Dual λ Absorbance Detector. The mobile phase was a mixture of 100 mM KH_2_PO_4_ (pH 3.5): Methanol (97:3, v/v).

Separations were performed on a C-18 column (Perfectsil Target ODS-3 (5 μM), 250´ 4.6 mm) with a C-18 guard column (Perfectsil Target ODS-3 (5 μM), 10´ 4 mm). The effluent was monitored by UV detection at 290 nm at a flow rate of 1.0 mL/min. Standard solutions of uric acid in the range of 0.1 to 20 mg/dL were prepared in mobile phase. Serum uric acid concentrations were expressed as mg/dL. 6-Mercaptopurine (1 mM) was used as the internal standard (21).

*XO and XDH activity determination*

The XO and XDH activity were measured spectrophotometrically by monitoring the production of uric acid from xanthine according to Prajda and Weber’s method (22). In the case of XDH, the assay mixture consisted of 50 mM phosphate buffer (pH 7.4), 200 µM NAD^+^, and 100 µL of the enzyme solution. After preincubation at 37°C for 15 min, the reaction was initiated by the addition of the substrate solution. After 30 min, the reaction was terminated by adding 0.5 mL HCl (0.6 M), and the absorbance was measured at 290 nm using a Shimadzu 2550 UV/VIS spectrophotometer which was controlled by the Shimadzu UV Probe personal software package including kinetics software. The instrument was connected to a Shimadzu cell temperature control unit. XO activity was measured using a similar method described for XDH with the difference being that molecular oxygen was used in place of NAD^+^ as electron acceptor. One unit (U) of activity was defined as 1 nmole of uric acid formed per min at 37°C, pH 7.4.

*Protein determination*

Protein concentration was determined spectrophotometrically by bicinchoninic acid kit using bovine serum albumin as the standard.

*Total antioxidant capacity assay*

The total antioxidant capacity of serum was determined by measuring its ability to reduce ferric ions (Fe^3+^) to ferrous form (Fe^2+^) by the FRAP (Ferric Reducing Ability of Plasma) test. The FRAP assay measures the change in absorbance at 593 nm owing to the formation of a blue colored Fe^2+^-tripyridyltriazine compound from Fe^3+^ by the action of electron donating antioxidants. The FRAP reagent consists of 300 μmole/mL acetate buffer (pH 3.6), 10 μmolee/mL tripyridyltriazine (TPTZ) in 40 μmole/mL HCl and 20 μmole/mL FeCl_3 _in the ratio of 10:1:1. Briefly, 30 μL of serum was added to 1.0 mL freshly prepared and prewarmed (37°C) FRAP reagent in a test tube and incubated at 37°C for 10 min. The absorbance of the blue colored complex was read against a reagent blank (1.0 mL FRAP reagent + 30 μL distilled water) at 593 nm. Standard solutions of Fe^2+ ^in the range of 10 to 1000 μmole/L were prepared from ferrous sulphate in water. FRAP values were expressed as μmole Fe^3+^ reduced to Fe^2+^ per liter (23).

*Lipid peroxide determination*

Lipid peroxide in the serum was measured using Yoshioka method (24). Briefly, 0.5 mL serum was shaken with 2.5 mL of 20% trichloroacetic acid (TCA) in a 10 mL centrifuge tube. 1 mL of 0.67% TBA was added to the mixture, shaken, and warmed for 60 min in a boiling water bath followed by rapid cooling. Then it was shaken into a 4 mL of *n*-butanol layer in a separation tube and malondialdehyde (MDA) content in the serum was determined at 532 nm by spectrophotometer against *n*-butanol. The standards of 0.1 to 20 μmole/L tetraethoxypropane (TEP) were used. The results were expressed as μmole/L serum.

*Statistical analysis*

All the samples and standards were run in duplicate and the results were expressed as mean ± SD. The statistical comparison between the experimental groups was performed by independent-sample t-test using SPSS computer program. The probabilities of 5% or less (p ≤ 0.05) were considered significant.

## Results and Discussion

Oral administration of parsley (*Petroselinum crispum*) and its major flavonol constituents (kaempferol and quercetin) for 14 days significantly reduced (p < 0.001) the serum uric acid levels of hyperuricemic but not normal rats. However, allopurinol treatment significantly reduced the levels of serum uric acid of both normal and hyperuricemic groups on day 14 (p < 0.01 and p < 0.001, respectively). The results also indicate that the parsley, kaempferol and quercetin exert their hypouricemic effects in a time-dependent manner in oxonate-pretreated rats (Table 1).

**Table 1:** Effect of the orally administered parsley, kaempferol and quercetin on serum uric acid levels (a time-dependent study).

**Groups**	**Uric acid (mg/dL)**		
	**Day 1**	**Day 7**	**Day 14**
Normal	1.65 ± 0.26^###^	1.62 ± 0.23^###^	1.68 ± 0.29^###^
Normal + parsley (5 g/Kg)	1.63 ± 0.25^###^	1.58 ± 0.54^###^	1.57 ± 0.48^###^
Normal + kaempferol (5 mg/Kg)	1.64 ± 0.26^###^	1.62 ± 0.18^###^	1.59 ± 0.18^###^
Normal + quercetin (5 mg/Kg)	1.65 ± 0.20^###^	1.61 ± 0.27^###^	1.59 ± 0.26^###^
Normal + allopurinol (5 mg/Kg)	1.52 ± 0.34^###^	1.37 ± 0.18^*^, ^###^	0.89 ± 0.41^**^ , ^###^
Hyperuricemic	3.49 ± 0.56^***^	3.54 ± 0.58^***^	3.61 ± 0.44^***^
Hyperuricemic + parsley (5 g/Kg)	3.35 ± 0.41^***^	2.87 ± 0.31^***^, ^#^	2.16 ± 0.31^*^, ^###^
Hyperuricemic + kaempferol (5 mg/Kg)	3.46 ± 0.51^***^	2.99 ± 0.42^***^	2.33 ± 0.38^**^, ^###^
Hyperuricemic + quercetin (5 mg/Kg)	3.20 ± 0.44^***^	2.83 ± 0.26^***^, ^#^	2.10 ± 0.30^*^, ^###^
Hyperuricemic + allopurinol (5 mg/Kg)	2.49 ± 0.23^***^ , ^##^	1.92 ± 0.43^###^	1.22 ± 0.32^*^, ^###^

All values are expressed as mean ± SD (n = 6). Independent-sample t-test was used for statistical significance assessment. ^*^indicates p ≤ 0.05, ^**^indicates p ≤ 0.01 and ^***^indicates p ≤ 0.001 *vs. *normal control group; ^#^ indicates p ≤ 0.05, ^##^ indicates p ≤ 0.01 and ^###^ indicates p ≤ 0.001 *vs. *hyperuricemic control group

The effects of the orally administered parsley (*Petroselinum crispum*) and its major flavonol constituents (kaempferol and quercetin) on liver XO/XDH activity after 14 days are summarized in Table 2. In oxonate-pretreated rats, all treatments resulted in significant inhibition on both liver XO and XDH activity. The reduction in liver XO and XDH activity in these hyperuricemic animals receiving parsley was 34.53% (p ≤ 0.01) and 43.71% (p ≤ 0.001), respectively. Kaempferol administration resulted in 31.32% (p ≤ 0.05) and 41.62% (p ≤ 0.001) inhibition on XO and XDH activity, respectively. Quercetin treated-hyperuricemic rats showed 34.53% (p ≤ 0.01) and 41.88% (p ≤ 0.001) inhibition on XO and XDH activity, respectively (Table 2).

**Table 2:** Effect of the orally administered parsley, kaempferol and quercetin on liver XO and XDH activity

**Groups**	**Activity (U/mg protein)**		**Inhibition %**	
	**XO**	**XDH**	**XO**	**XDH**
Normal	2.50 ± 0.63	3.80 ± 0.38	-	-
Normal + parsley (5 g/Kg)	2.30 ± 0.70	2.93 ± 0.37^**^ , ^#^	8.00	22.89
Normal + kaempferol (5 mg/Kg)	2.39 ± 0.26	3.07 ± 0.42^*^ , ^#^	4.40	19.21
Normal + quercetin (5 mg/Kg)	2.32 ± 0.31	3.15 ± 0.46^*^	7.20	17.10
Normal + allopurinol (5 mg/Kg)	1.17 ± 0.28^***^ , ^###^	1.47 ± 0.40^***^ , ^###^	53.20	61.31
Hyperuricemic	2.49 ± 0.52	3.82 ± 0.62	-	-
Hyperuricemic + parsley (5 g/Kg)	1.63 ± 0.29^*^, ^##^	2.15 ± 0.26^***^ , ###	34.53	43.71
Hyperuricemic + kaempferol (5 mg/Kg)	1.71 ± 0.37^*^, ^#^	2.23 ± 0.51^***^ , ###	31.32	41.62
Hyperuricemic + quercetin (5 mg/Kg)	1.63 ± 0.39^*^, ^##^	2.22 ± 0.38^***^ , ###	34.53	41.88
Hyperuricemic + allopurinol (5 mg/Kg)	1.05 ± 0.18^***^ , ^###^	1.27 ± 0.33^***^ , ^###^	57.83	66.75

All values are expressed as mean ± SD (n = 6). Independent-sample t-test was used for statistical significance assessment. ^*^indicates p ≤ 0.05, ^**^indicates p ≤ 0.01 and ^***^indicates p ≤ 0.001 *vs. *normal control group, ^# ^indicates p ≤ 0.05, ^##^ indicates p ≤ 0.01 and ^###^ indicates p ≤ 0.001 *vs. *hyperuricemic control group

**Figure 1:**
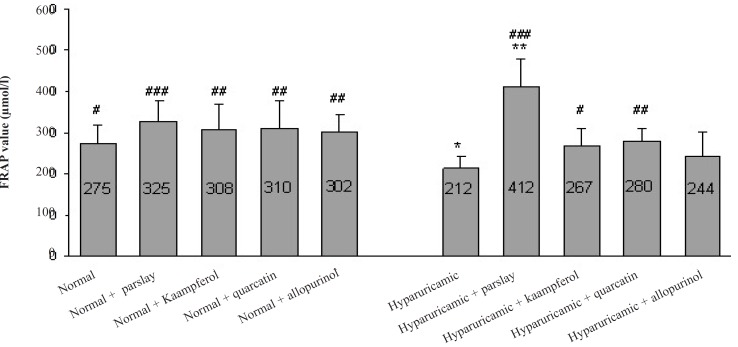
Effect of the orally administered parsley, kaempferol and quercetin on serum total antioxidant capacity (FRAP value) after 14 days (mean ± SD, n = 6). ^*^ indicates p ≤ 0.05 and ^**^ indicates p ≤ 0.01 *vs. *normal control group, ^#^ indicates p ≤ 0.05, ^## ^indicates p ≤ 0.01 and ^###^ indicates p ≤ 0.001 *vs. *hyperuricemic control group

In hyperuricemic control rats, the total antioxidant capacity was significantly lower than that of normal rats and following treatment of these animals with parsley a significant increase (p ≤ 0.001) in total antioxidant capacity was observed. Kaempferol and quercetin treatment was also able to increase total antioxidant capacity in huperuricemic rats significantly (p ≤ 0.05 and p ≤ 0.01, respectively) (Figure 1).

**Figure 2:**
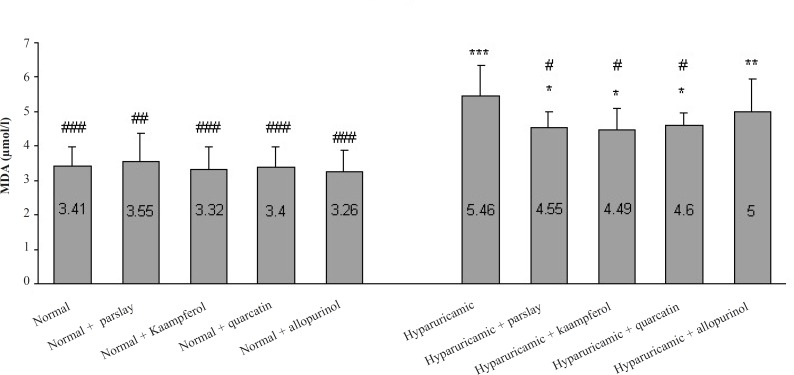
Effect of the orally administered parsley, kaempferol and quercetin on MDA concentration after 14 days (mean ± SD, n = 6). ^*^ indicates p ≤ 0.05, ^**^ indicates p ≤ 0.01 and ^***^ indicates p ≤ 0.001 *vs. *normal control group, ^#^ indicates p ≤ 0.05, ^## ^indicates p ≤ 0.01 and ^### ^indicates p ≤ 0.001 *vs. *hyperuricemic control group

In hyperuricemic control rats, the levels of serum MDA, as a biomarker of lipid peroxidation, were statistically higher than those of normal rats (p ≤ 0.001). Oral administration of parsley, kaempferol and quercetin to hyperuricemic rats induced a significant reduction (p ≤ 0.05) in these elevated levels of MDA after 14 days, but could not yet reach these levels to the normal value (Figure 2).

Based on the results, the oral administration of parsley (*Petroselinum crispum*)*, *kaempferol and quercetin exert notable hypouricemic effects in hyperuricemic but not in normal rats. In contrast, allopurinol reduced serum uric acid levels of both normal and hyperuricemic rats and the levels even reached to the level lower than that of normal values. These results indicate that parsley (*Petroselinum crispum*) and its major flavonol constituents (kaempferol and quercetin) might bring fewer side effects than allopurinol in treatment of hyperuricemia. On the other hand, this property of parsley (*Petroselinum crispum*) and its major flavonol constituents (kaempferol and quercetin) could be considered as an advantage for this medicinal plant and its flavonol constitutes. Although the elevated levels of uric acid in the circulation could give rise to gout and possibly other pathological conditions (25), the antioxidant action of uric acid, particularly its ability to inhibit DNA damage, is also well documented (25, 27). Thus, excessive lowering of the uric acid level in the circulation beyond that of the normal range might even be counterproductive (27). Kong *et al. *have also shown that the water extract of Ermiao wan (a Chinese herbal medicine used in the treatment of acute gout) have less inhibitory effects on serum uric acid levels in normal mice compared with those animals pretreated with potassium oxonate (18). Taking into account that parsley (*Petroselinum crispum*) as a dietary vegetable can be used safely long-term; this feature of parsley makes it a possible alternative for allopurinol, or at least in combination therapy to minimize the side-effects of allopurinol.

The results also indicate that parsley, kaempferol and quercetin exert their hypouricemic effects in a time-dependent manner in oxonate-pretreated rats. In parsley treated-hyperuricemic rats, uric acid reduction was not statistically significant on the first day, but after 7 and 14 days of intervention, a significant (p ≤ 0.05 and p ≤ 0.001, respectively) reduction was observed in the uric acid levels of hyperuricamic rats. Similar results was also observed in quercetin treated- hyperuricemic rats. In kaempferol treated-hyperuricemic rats, only after 14 days of intervention, serum uric acid levels reduced significantly (p ≤ 0.001) compared to hyperuricemic control rats. Unlike these, the hypouricemic effect of allopurinol was statistically significant (p ≤ 0.01) even after 1 day of the drug administration indicating the quicker onset of allopurinol action compared to that of parsley, quercetin and kaempferol.

The inhibitory effect of parsley (*Petroselinum crispum*) and its major flavonol constituents (kaempferol and quercetin) on liver XO/XDH activity was also confirmed in this study. XO/XDH is the key enzyme in the catabolism of purines and has a critical role in the endogenous production of uric acid (5). Several *in-vitro *studies confirmed the XO/XDH inhibitory activity of some flavonoids. These compounds are structurally similar to XO/XDH substrate and so can inhibit the enzyme activity (1, 7). Therefore the hypouricemic property of parsley, kaempferol and quercetin, observed in this study, could be explained at least in part by the inhibitory effects of them on XO/XDH activity. The extent of reduction in XO/XDH activity elicited by allopurinol was much higher than that observed with the parsley, kaempferol and quercetin administration in both normal and hyperuricemic groups. Similar results have been reported by others (18-21). According to these studies, the involvement of other possible mechanisms such as enhanced uric acid clearance or actions on other purine metabolizing enzymes cannot be ruled out (18, 19). This could be further supported by the existence of some hypouricemic compounds including natural products that are devoid of XO/XDH inhibitory activity (18, 20, 27). It seems that the inhibitory effects of parsley, kaempferol and quercetin on XO/XDH activity in hyperuricemic rats are more dominant than their effects on the normal activity of either two forms of the enzyme (Table 2). Similar results have been reported by Zhao *et al. *(19). It is well known that XO and XDH are inducible enzymes and oxonic acid and its salt might induce XO and XDH activity in liver (28). However, our results could not explain why oxonate did not appear to increase the enzyme activity.

In addition, in this investigation we observed a significant increase in serum total antioxidant capacity and decrease in MDA concentration, following treatment of the hyperuricemic rats with parsley, kaempferol and quercetin. It is worth to note that parsley, kaempferol and quercetin exerts mostly their antioxidant effects in hyperuricemic groups rather than in normal groups. Several studies have identified the active antioxidants within parsley (*Petroselinum crispum*) including flavonoids (14), carotenoids (29), ascorbic acid (30), tocopherol and coumarines (14). These phytochemicals improve total antioxidant capacity, suppress destructive oxygen free radicals and prevents oxidative stress damage (10, 31). Our study also showed that parsley (*Petroselinum crispum*) has a higher potential than its major flavonol constituents to increase serum total antioxidant capacity. In parsley, kaempferol and quercetin glycosides are predominant, and it is well known that the bioavailability of flavonoids as glycosidic form is higher than that of aglycon form, therefore; this finding could be attributed to its higher intestinal absorption. However, the type of glycosidic bound is different from one flavonoid to other and it may affect on its absorpsion (32). In this study, allopurinol treatment could not significantly compensate the abased total antioxidant capacity or the elevated level of MDA concentration in hyperuricemic rats. However, the inhibition of XO by allopurinol was previously reported to decrease the level of ROS production and reduce the hepatic injury associated with liver transplantation (33).

## Conclusion

Parsley (*Petroselinum crispum*) and its major flavonol constituents are capable of reducing the uric acid levels in hyperuricemic rats with no effects on the level of this biological metabolite in normal animals and prevent oxidative stress. Such hypouricemic effects may be attributed, at least in part, to XOR inhibitory action of them. Therefore, the use of suboptimal dosages of allopurinol in combination with parsley intake may provide a safer approach for prevention and treatment of hyperuricemia. Further investigations to explore the effect of other components of parsley (*Petroselinum crispum*) and define their clinical efficacy would be highly desirable. 
